# Evaluation of Pozzolanic and Alkali-Activated Reactivity of Low-Purity Calcium Bentonite

**DOI:** 10.3390/ma15228015

**Published:** 2022-11-14

**Authors:** Wanqiang Li, Chunmeng Jiang, Qin Zhang, Shuangxi Li

**Affiliations:** 1 College of Water Conservancy and Civil Engineering, Xinjiang Agricultural University, Urumqi 830052, China; 2 Xinjiang Key Laboratory of Hydraulic Engineering Security and Water Disasters Prevention, Urumqi 830052, China

**Keywords:** alkali-activated, calcium bentonite, pozzolanic activity, C-S-H gels

## Abstract

Alkali-activated cement (AAC) is a sustainable building material with low carbon emissions, but it has a growing demand for raw materials. In this study, the potential of low-purity modified calcium bentonite (CB) as a raw material for AAC was evaluated. The thermodynamic changes and pozzolanic properties of calcined CB were determined using X-ray diffraction (XRD), thermogravimetry-differential thermal analysis (TG-DTA), zeta potential, and a strength activity index (SAI) test. The compressive strength test, scanning electron microscopy–energy dispersive spectrometer (SEM-EDS), and Fourier-transform infrared (FTIR) spectroscopy were performed to examine the compatibility between CB and AAC. It was revealed that CB is a low-purity clay with low-pozzolanic activity. Calcination enhanced its pozzolanic activity, and the optimum temperature is 750 °C. The incorporation of modified CB improved the mechanical properties of AAC, and low-temperature modified CB had better compatibility with AAC than the high-temperature modified CB. Calcination at 150 °C had little effect on the structure of CB, and the water absorption of montmorillonite increased the ion concentration, increasing the rate and degree of hydration. Furthermore, low-temperature calcination had a dissolution–precipitation effect, resulting in leaf-like CaO·SiO_2_·H_2_O (C-S-H) gels, whereas the high-temperature calcination of CB was very reactive, resulting in flower-like C(N)-S-H gels.

## 1. Introduction

Since its invention in 1824, cement has emerged as an essential building material due to its high early strength and excellent performance [1]. However, a significant amount of CO_2_ is generated during cement production, accounting for 7% of global emissions [2]. Owing to the high energy consumption and carbon emissions of cement, considerable effort has been devoted to identifying a sustainable and green solution to counter these problems.

Supplementary cement material (SCM) is considered an effective method for reducing cement production. The principle of SCM is to consume the calcium hydroxide produced via cement hydration through a pozzolanic reaction to generate additional CaO·SiO_2_·H_2_O (C-S-H) gels that fill the hydrated pores and improve the performance of cement [3,4]. However, controlling the secondary hydration reaction in cement is complicated. Greater emphasis must be placed on the quality of the raw material. This limitation is more significant when low-purity clays are used to prepare the SCM [5]. Alkali-activated cement (AAC) is a clinker-free cement; its raw materials can be slag [6], fly ash [7], metakaolin [8], and other industrial wastes or nanoclays [9]. AAC has the advantages of fast hardening, early strength, corrosion resistance, and fire resistance [10,11]. More importantly, its carbon emissions are only 20% of ordinary Portland cement, which is considered a green building material [12]. The principle mechanism of AAC is that the raw materials depolymerize under the actions of alkali metal ions and OH^−^, and the ionic bonds of the silicon–oxygen tetrahedron and aluminum–oxygen polyhedron are broken to form silicate and aluminate monomers [13]. The monomers undergo a polycondensation reaction and gelation, precipitating water and polymerizing into amorphous aluminosilicate gels and zeolite crystals [10]. Directly providing the alkaline environment required for the reaction appears to be more efficient for comparing the two reaction principles, suggesting that the preparation of AAC using low-pozzolanic clay is more suitable than that of SCM.

Bentonite is composed of two layers of silicon–oxygen tetrahedra and one layer of aluminum–oxygen octahedra, and its main component is montmorillonite. Montmorillonite contains a wide and variable interlayer spacing, which allows for the interchange of ions (e.g., Na^+^, Ca^2+^) between the layers. It has absorbent swelling properties and can absorb water 8–15 times its volume [14]. Bentonite has unique properties and compatibility that can be modified using physical and chemical methods. For example, bentonite has low pozzolanic activity, and high-temperature calcination removes the hydroxyl groups in its structure to improve reactivity [15]. The ion layer spacing of acid-treated bentonite increases, and the bentonite exhibits a weak adsorption capacity [16]. Under alkaline conditions, the cations in montmorillonite depolymerize to form hydroxylated monomers and dimers. When the pH of the solution decreases, the dissolved montmorillonite and silica can serve as templates for the precipitation of aluminosilicates and other minerals, resulting in polymerized hydroxyl structures [17,18]. Numerous studies have shown that bentonite treated in highly alkaline solutions exhibits enhanced ion exchange and that dissolution–precipitation of montmorillonite generates zeolites and C-S-H gels [19,20]. The successful preparation of AAC using bentonite has been reported, but only a few studies have been conducted on low-purity calcined clay [21,22,23].

Compared to other types of clay, bentonite has storage and performance advantages. However, studies on the pozzolanic activity of bentonite have been under-appreciated. Using bentonite as an SCM is inefficient, and its role in hydration is ambiguous and unfavourable. Therefore, calcined bentonite has rarely been studied as an AAC material, and there is no correlation between the pozzolanic and alkali-activated activity. In this study, the correlation between the properties of low purity calcium bentonite (CB) and calcination temperature was investigated, and the effects of CB on hydration and hydration products were examined. The suitability and potential of CB as a gelling material were characterized by pozzolanic and alkali-activated activity. This study may provide insights for expanding the application of CB as a construction material.

## 2. Experimental Sections

### 2.1. Materials

The chemical components of slag, fly ash, cement, and CB were determined using X-ray fluorescence (XRF, ARL PERFORM’X model), and the results are listed in Table 1.

The slag grade is S75, the fly ash is low-calcium ClassII F, and the cement is Portland benchmark cement; all of them were provided by the Yuance Co. (Changji, China). The CB used was a low-purity, untreated raw mineral provided by Xiangshui Building Materials Co. (Yueyang, China). Water glass and flake sodium hydroxide were provided by Jinxin Factory (Urumqi, China). The SiO_2_/Na_2_O of water glass is 3.0, and flaky sodium hydroxide purity >99%.

### 2.2. Treatment and Activity Assessment for CB

#### 2.2.1. Thermal Treatment

Initially, a muffle furnace and crucible were heated to a constant temperature (set to normal temperature, 150 °C, 300 °C, 450 °C, 600 °C, 750 °C, 900 °C), and CB was then placed in the crucible and calcined in the muffle furnace for 2 h. Subsequently, the calcined CB was placed in a drying dish to prevent moisture adsorption, left until completely cooled, and stored in a sealed bag.

#### 2.2.2. Phase Analysis of CB

The thermogravimetry-differential thermal analysis (TG-DTA, STA 449F3 model) of CB was performed in a temperature range of 30–1000 °C at a heating rate of 10 °C/min in air. X-ray diffraction (XRD, X’PertPRO model) was conducted with CuKα radiation at a scanning speed of 2°/min, a voltage of 40 kV, and a scanning range of 10–50°.

#### 2.2.3. Zeta Potential

Sieved CB (0.1 g) was mixed with water (99.9 g) at a ratio of 1000:1 to prepare a colloidal suspension. The suspension was stirred with a magnetic bar for 5 min before the analysis to prevent colloidal aggregation and precipitation. The zeta potential was measured (DT-310 model) at a data collection interval of 2 min, and the results from three measurements were averaged and recorded. All the measurements were conducted at room temperature.

#### 2.2.4. Evaluation of Pozzolanic Activity

Pozzolanic activity evaluations were performed using the strength activity index (SAI) method. According to the Chinese standard (GB/T 12957-2005 test method for the activity of industrial waste slag used as an addition to cement), the total mass of the mixture was 450 g, and 70% of the benchmark cement was mixed with 30% calcined CB and added to standard sand at a water-to-binder ratio of 0.5 to prepare a 40 × 40 × 160 mm mortar bar. A 100% cement control group was prepared and cured under standard conditions for 28 d, and its compressive strength was tested. The SAI test result was calculated as the ratio of the compressive strength of the same age blended cement mortar (A) to that of the control group (B), expressed as a percentage (SAI = A/B × 100). The Chinese standard indicates that a material with a pozzolanic activity greater than 75% SAI after 28 days is satisfactory for this test.

### 2.3. Preparation: Strength Measurement and Characterization of AAC

#### 2.3.1. Compressive Strength

For compressive strength analysis, water glass and sodium hydroxide were mixed in specific proportions, and the water glass modulus was adjusted to 1.4 and cooled to room temperature before use. In addition, the total mass of the mixture was 450 g, in which 65% slag, 20% fly ash, and 15% calcined CB were mixed in a planetary cement mortar in proportion, water and 5% water glass (calculated as N_2_O wt%) were added at a water-to-binder ratio of 0.5 (the water in the water glass was deducted in advance). Next, the sample was vibrated and shaped in a steel mould, covered with a layer of cling film to prevent moisture loss, and placed in a curing oven at 20 ± 1 °C and humidity greater than 95% for 28 d to test the compressive strength. The preparation was based on the Chinese standards (GB/T 17671-1999 Method of testing cements–Determination of strength ISO method).

#### 2.3.2. Microstructural Investigation of AAC

The samples of the scanning electron microscopy–energy dispersive spectrometer (SEM, Supra55 VP model) were immersed in anhydrous ethanol to terminate the hydration process. The SEM in this test had a magnification of 150–20,000×, an accelerating voltage of 20 kV, a working distance of 8.0–10.8 mm, and an SE2 detector. The sample was dried in a constant temperature blast oven at 50 °C for 24 h until a constant weight was obtained, placed in an agate grinding bowl, finely ground, and passed through a 75 µm standard sieve until there was no visible grain, with a texture similar to that of flour. Fourier-transform infrared (FTIR, Nicolet iS20 model) spectroscopy was performed in the range of 4000–400 cm^−1^, and the test samples were pre-treated in a manner similar to the SEM characterization. 

## 3. Results and Discussion

### 3.1. Thermodynamic Changes of CB

**Figure 1:**
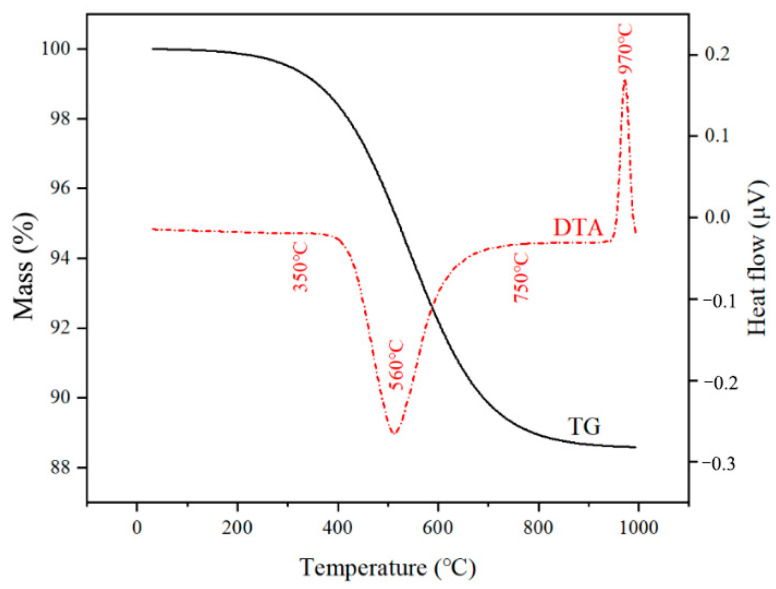
TG-DTA curve of CB.

The TG-DTA curves of CB are shown in Figure 1.

The TG curve represents the mass loss of CB during heating, whereas the DTA curve represents the heat absorption and exothermic behaviour during heating. From the figure, the calcination process of CB can be divided into four stages. In the first stage, the mass loss occurs mainly in the range of 30–350 °C, where the CB absorbs heat and removes adsorbed water and interlayer water [24]. Based on the TG-DTA curves, there was a clear difference in the rate of heat absorption between the adsorbed water and interlayer water losses. The higher the degree of collapse of the interlayer space of montmorillonite, the higher is the interlayer water loss rate. The complete removal of water results in changes in the surface area and water absorption behaviour of montmorillonite [25]. The mass loss rate increased between 350 and 560 °C (second stage). The mass loss at this stage was derived from the evaporation of secondary mineral hydroxyl water [24]. The third stage of mass loss occurred in the range of 560–750 °C. A large and prominent heat absorption peak can be observed, and the temperature of the heat absorption peak decreases as the degree of disorder is negligible. For instance, the lattice structure of bentonite calcined at 560 °C shows less disorder [26]. As the temperature increases, the lamellar structure collapses, hydroxyl water overflows, and the physicochemical properties of montmorillonite change with the loss of water absorption and ion exchange properties [27]. In the fourth stage (750–900 °C), the mass loss of CB gradually stabilized and the dehydroxylation rate decreased, which is indicated by the sharp upward exothermic peak. In this temperature range, montmorillonite and quartz undergo recrystallization reactions, and spinel phases are formed [28].

### 3.2. Crystal Phase Change of CB

**Figure 2:**
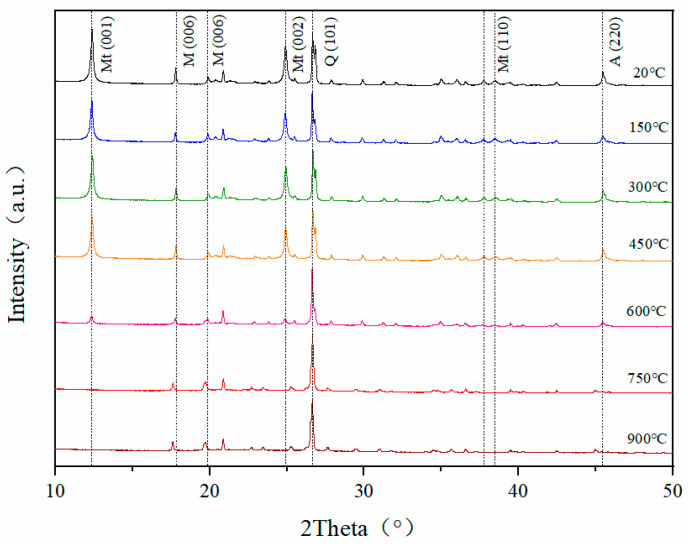
XRD pattern of CB (Mt—montmorillonite, M—muscovite, Q—quartz, A—albite).

Uncalcined CB (Figure 2) contains a substantial amount of montmorillonite (001, PDF#13-0135), the predominant impurities are muscovite (006, PDF#46-1409), quartz (101, PDF#46-1045) and a small amount of albite (220, PDF#89-6425).

In the calcination temperature range of 150–450 °C, the height of the diffraction peaks remained constant, but the width became narrower. Combined with the TGA data, it can be speculated that the disappearance of interlayer and adsorbed water causes the change in the diffraction peak width, and the heat treatment at 450 °C is insufficient to convert montmorillonite to the dehydroxylated state [29]. Unlike the results of low-temperature calcination, the diffraction peaks of montmorillonite decreased significantly for bentonite calcined at 600 °C. At 750 °C, the diffraction peaks of montmorillonite disappeared completely. It indicates that the dehydroxylation reaction of montmorillonite occurred and the amorphous aluminosilicate phase was formed [30,31]. At 900 °C, the change in the quartz diffraction peak at 26.5° indicates that the amorphous structure has undergone recrystallization and the structure is gradually stabilized, which is in agreement with the TG-DTA results [32,33].

### 3.3. Zeta Potential of CB

The zeta potential is closely related to particle dispersion in the sample. The higher the zeta potential, the better the stability, and the lower the aggregation. Due to the lattice disruption and isomorphic substitution, montmorillonite is usually significantly electronegative in solution [34]. When montmorillonite particles come into contact with water, a double layer forms between the particle surface and electrolyte to balance the negative charge. Counter ions in the solution are tightly adsorbed on the particle surface, forming a Stern layer. Subsequently, the counter ions outside the Stern layer were distributed in the solution via diffusion, forming a diffusion layer [35,36]. When montmorillonite is suspended in a solution, the interlayer ions can be replaced by alkali metal cations. Therefore, the zeta potential values can be used to examine changes in the ion exchangeability of montmorillonite during calcination and to determine the effect of changes in clay stability on pozzolanic activity.

**Figure 3:**
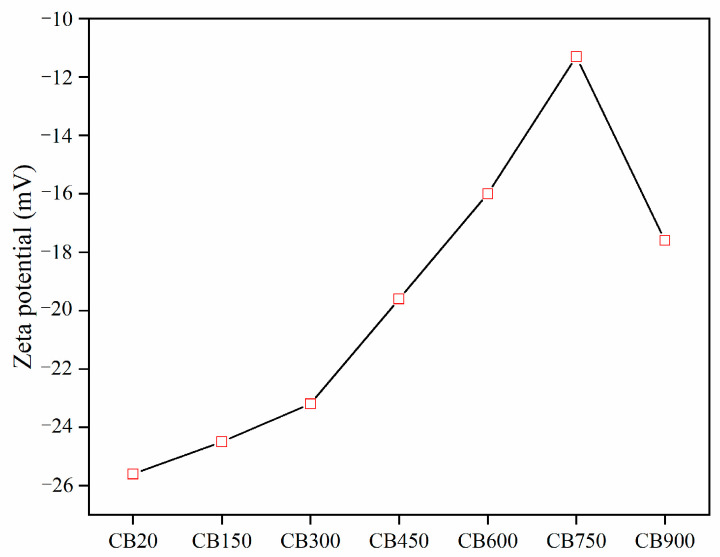
Zeta potential of CB.

At room temperature, an adequate dispersion of CB in the solution was observed, showing a strong negative potential (Figure 3).

The decrease in zeta potential in the range of 150–300 °C is attributed to the loss of the interlayer water of CB, which results in a smaller layer spacing, enhanced ion exchange capacity of montmorillonite, increased adsorption capacity, and reduced electronegativity [37]. At 300–600 °C, the change in zeta potential was attributed to the loss of interlayer water, and the highest ion adsorption capacity was attained. 

The loss of interlayer water leads to the direct exposure of the montmorillonite surface to high temperatures. As H_2_O molecules are neutral, the surface potential does not change. However, these sites are more readily bound to protons or other cations in solution owing to the reduced resistance. At 600–900 °C, the zeta potential of CB increased and then decreased, which is consistent with the overall transition of montmorillonite from dehydroxylation to recrystallization. In this temperature range, the structure of montmorillonite is disrupted, and the adsorption capacity and electronegativity are lost. In addition, proton transfer occurs between neighbouring hydroxyl groups, and water is eliminated at higher temperatures. The active Si-O-H bonds break and recombine to form the stronger Si-O-Si bonds. Furthermore, increases in the amorphous aluminosilicate content reduced the cohesiveness and increased the zeta potential [38]. When the calcination temperature was more than 900 °C, CB underwent a recrystallization reaction, restoring its crystalline characteristics and decreasing the zeta potential. 

### 3.4. SAI Analysis

**Figure 4:**
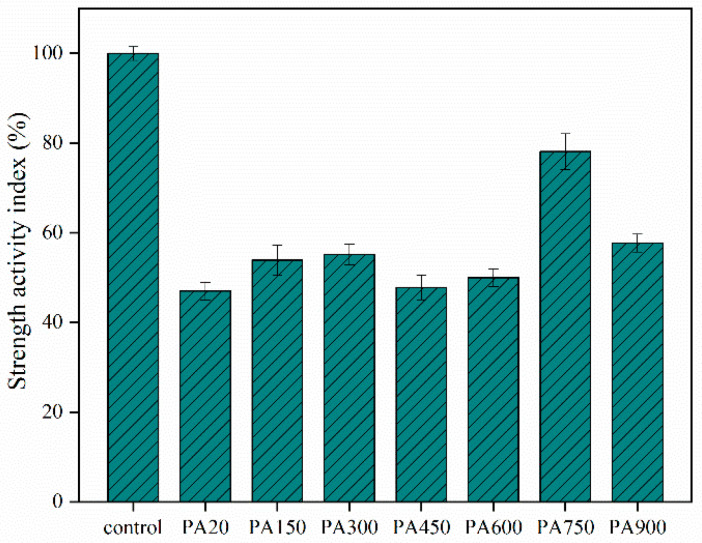
Strength activity index of calcined CB.

Figure 4 shows the results of the SAI test, which can reflect the pozzolanic activity of the calcined CB.

CB exhibits the lowest pozzolanic activity at room temperature. As the calcination temperature increases, the activity initially increases and then decreases. The free water of CB evaporates under low-temperature calcination, and montmorillonite adsorbs water from the solution in the initial stages of hydration, indirectly increasing the ion concentration and accelerating the hydration reaction. Based on the XRD results, it is clear that the CB sample starts to remove the hydroxyl groups from its structure at 600 °C, and the CB is in a state of simultaneous ion-exchangeability and pozzolanic activity.

On the one hand, ion exchangeability requires the adsorption of cations to reach charge equilibrium. On the other hand, the pozzolanic activity requires a reaction in the presence of alkali metal ions. Generally, the ion adsorption rate of amorphous silica-aluminates is much higher than the pozzolanic reaction. Some of the reaction products cannot function effectively, even if the montmorillonite is attacked by alkaline solutions, releasing the adsorbed cations, and it is difficult to compensate for the loss of compressive strength. The maximum strength index reached a peak of 78% at 750 °C, and the CB reached the SCM use standard. At this temperature, montmorillonite loses all its properties and completely transforms into an amorphous state with the highest reactivity. At a calcination temperature of 900 °C, the pozzolanic activity was limited by recrystallization. Since the dehydroxylation reaction in CB is terminated, new crystal phases are formed, enhancing structural stability. 

Therefore, based on the test results, 750 °C was the best pozzolanic activation temperature for CB. 

**Figure 5:**
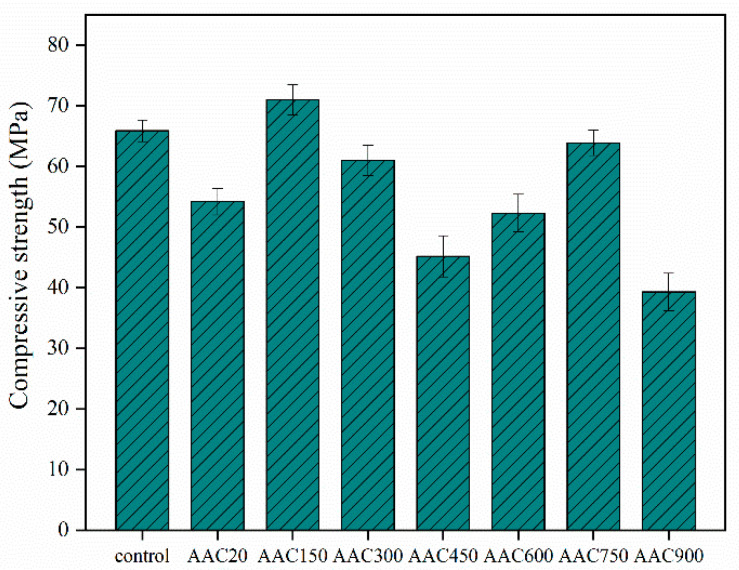
28 days compressive strength of the AAC.

The incorporation of uncalcined CB reduced the compressive strength of AAC (Figure 5). The compressive strength peaked at 71 MPa at a calcination temperature of 150 °C. As the temperature increases, the compressive strength decreases, reaching a second peak at 750 °C. At 900 °C, the compressive strength value decreases sharply.

The increase in the compressive strength of the AAC at 450–900 °C can be attributed to an increase in the degree of dehydroxylation in CB, which is consistent with the results of the pozzolanic activity tests. The dehydroxylation of montmorillonite produces reactive components, such as SiO_2_ and Al_2_O_3_, which can react with bases in alkaline environments to form C-S-H gels [39,40]. However, the stable crystalline phase structure reduced the CB reactivity and compressive strength.

The optimum alkali activation temperature for CB is 150 °C. The difference in the calcined strength and pozzolanic activity between 150 °C and 300 °C is attributed to water absorption. At this temperature, CB did not undergo dehydroxylation reactions, the structure was not significantly changed, and the chemical bonds were maintained. However, the adsorbed interlayer water was removed from the structure. CB adsorbed the surrounding free water and other reactants in the interlayer during the initial stages of the reaction [23]. The reduction in free water leads to an increase in the ion concentration and a faster reaction rate. Ca^2+^ in the interlayer is exchanged with higher concentrations of Na^+^, releasing Ca^2+^ and generating C-S-H gels under the pozzolanic reaction conditions, thereby increasing the compressive strength of the AAC [22,41]. In the later stages of the reaction, dissolved montmorillonite loses its ion exchange and water absorption properties and participates as a reactant in the formation of hydration products, which have an excellent filling effect on the pores within the structure, resulting in a more compact hydrated structure and higher compressive strength of the AAC.

### 3.5. Compressive Strength of AAC

#### 3.5.1. SEM Characterization

**Figure 6:**
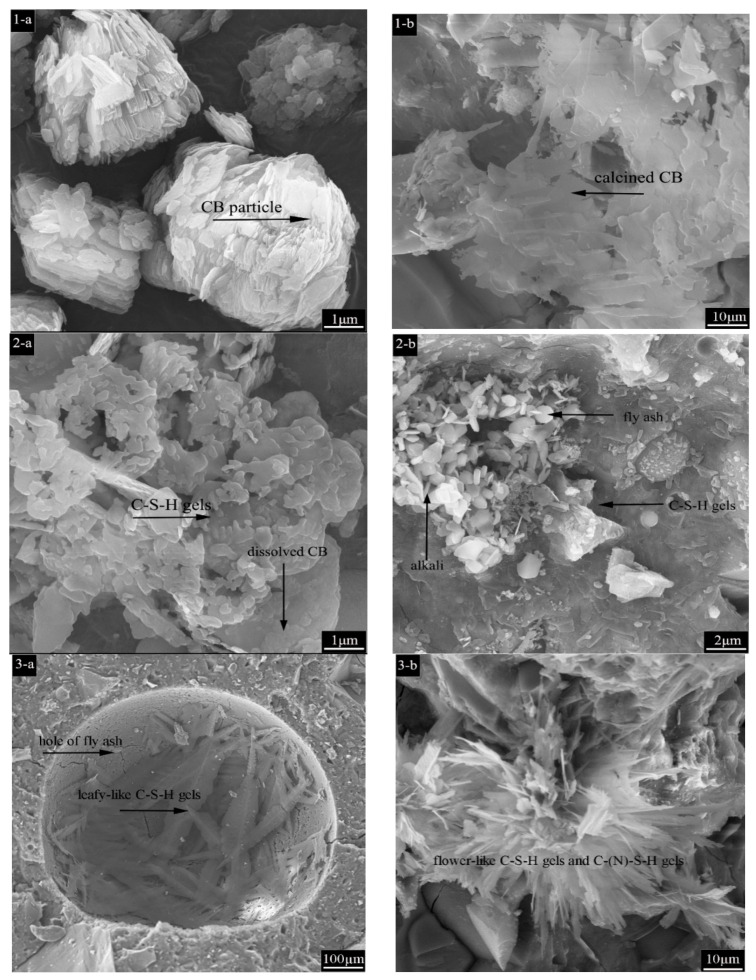

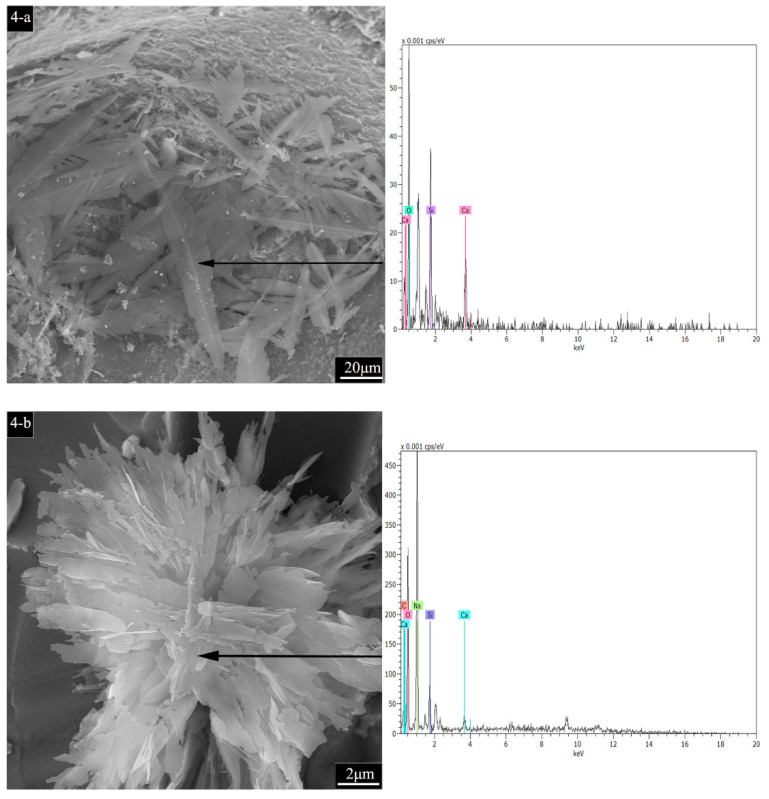
(**1-a**) CB particles at 20 °C, (**1-b**) CB after calcination at 750 °C for 2 h. (**2-a**) Hydrated morphology of CB particles with C-S-H gels at 20 °C, (**2-b**) Hydrated morphology of CB particles with C-S-H gels after calcination. (**3-a**) Leaf-like gels in AAC150 sample, (**3-b**) Flower-like gels in AAC750 sample. (**4-a**) EDS elemental analysis of the leaf-like gels, (**4-b**) elemental analysis of the flower-like gels.

The layered montmorillonite structure consists mostly of irregular sheets stacked together (Figure 6(1-a)), with a certain distance between the sheets to allow for cation exchange.

Upon heat treatment, the structure collapsed, the stacked matrix dispersed and disintegrated, and montmorillonite no longer retained its crystalline structure (Figure 6(1-b)). Dissolved montmorillonite binds more tightly to the C-S-H gel, generating structures without distinct cracks or pores, and CB participates as a reactant in the hydration reaction of AAC to produce the corresponding hydration products (Figure 6(2-a)). Nevertheless, non-layered montmorillonite structures in calcined CB were replaced by C-S-H gels and flaky alkali components (Figure 6(2-b)). Calcination at a high temperature causes montmorillonite in CB to lose its ability to absorb water, allowing it to be utilized only as a filler without altering the pace of hydration reactions, which result in the partial hydration of the fly ash. According to the EDS results, the pores of AAC150 were filled with leaf-like gels, which are C-S-H gels produced by dissolved fly ash (Figure 6(3-a),(4-a)). Furthermore, foliated gels were observed in AAC750 instead of flower-like zeolite structures, which indicates the presence of C (N)-S-H gel precursors, according to the EDS results (Figure 6(3-b),(4-b)). The presence of coexisting gels was due to the lower solubility of Na^+^ replacing Ca^2+^, resulting in a low Ca/Si ratio, with a molecular formula of Na_2_O-CaO-SiO_2_-H_2_O [42]. This confirms that CB calcined at 150 °C reacts more rapidly with alkali than that calcined at 750 °C at the same stage, improving the AAC hydration process and enhancing the compressive strength.

#### 3.5.2. FTIR Characterization

**Figure 7:**
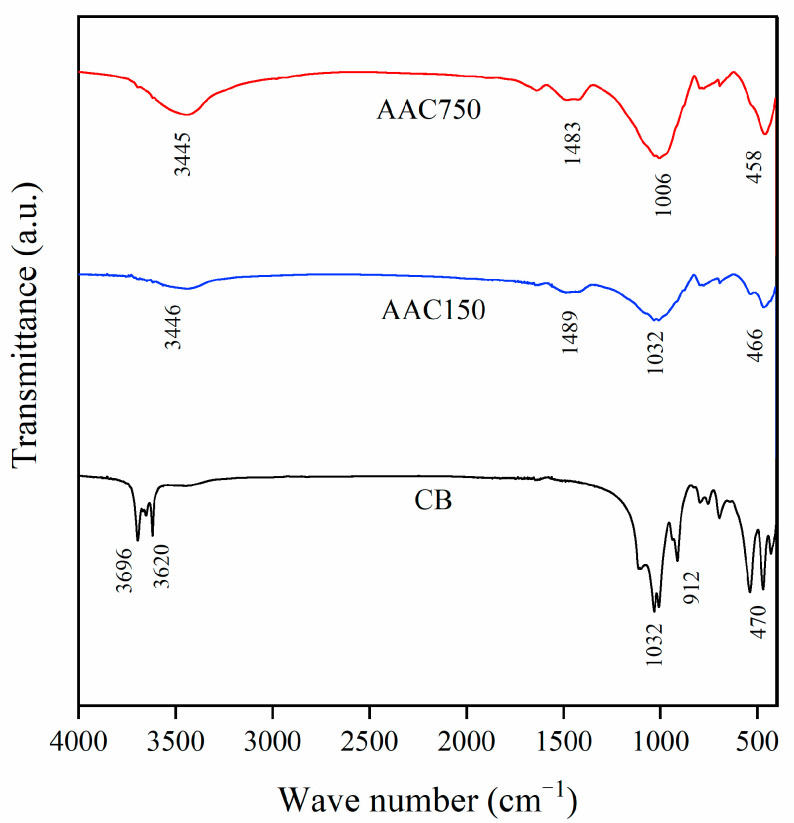
FTIR spectra of CB and the AAC.

There are four distinct transmission peaks (Figure 7) in the 3700–3600 cm^−1^ region which belong to the stretching vibrations of the O-H crystal bands in the silicon–oxygen tetrahedra and aluminum–oxygen octahedra.

The position and amplitude of the O-H peaks change due to coordination cations, and the four peaks are considered to be in an ordered state [43]. The peaks corresponding to the Si-O stretching and O-H bending vibrations of CB fall in the 1400–400 cm^−1^ range. The strong band at 1030 cm^−1^ is ascribed to Si-O stretching vibrations, whereas the peak at 912 cm^−1^ correlates with the Si-O asymmetric stretching motion and belongs to the crystalline solid band of the double octahedral mineral, indicating the position of the hydroxyl group bound to the two octahedrally coordinated cations (Al^3+^ or Mg^2+^) in the montmorillonite structure [44]. The strong band at 470 cm^−1^ is attributed to the Si-O-Si bending vibrations in the tetrahedral silicon [45]. For the AAC samples, the shape of the AAC150 peaks at 466 cm^−1^ and 1032 cm^−1^ is similar to that of CB, showing no change in the shape and position of the bands but a decrease in intensity, indicating that CB can maintain the hydroxyl structure under alkaline conditions, but some of the structures are still involved in the hydration reaction as reactants. The bands at 1489 cm^−1^ and 3446 cm^−1^ were attributed to O-H stretching vibrations. The position of the FTIR peak of AAC750 was shifted more significantly, and its intensity was higher compared to that of AAC150. The intensity of the peak at 3446 cm^−1^ was lower than that at 3445 cm^−1^ because the low-temperature calcined CB had higher water absorption capacity, resulting in slightly higher water content and lower peak intensity. The most prominent peak in the sample is observed at 1006 cm^−1^, which is ascribed to the asymmetric stretching vibration of Si-O-T (T is either Si or Al), and the amplitude of the vibration is Si/Al dependent [22,46]. The Si/Al molar ratio in the AAC750 is significantly higher than that in AAC150, which is related to the pozzolanic activity at elevated calcination temperatures, increasing the amount of active SiO_2_. Accordingly, CB that retains its water absorption capacity is more suitable for reaction with alkaline solutions than thermally activated CB, which causes the AAC compressive strength to fluctuate and then increase.

In summary, the CB calcined at 150 °C and 750 °C fulfilled the AAC specifications, and the CB calcined at 150 °C had more advantageous and required fewer treatment steps to improve its performance, resulting in lower energy consumption and carbon emissions.

## 4. Conclusions

The focus of this study was to evaluate the pozzolanic activity of low purity CB before and after calcination as well as its potential when used as a raw material for the AAC to reduce cement production and carbon emissions. Based on the results, the following conclusions were drawn:

1. Calcination can change the pozzolanic activity of CB. In the temperature range of 150–560 °C, CB only removes water and does not change its properties. At 560–900 °C, CB underwent dehydroxylation and then recrystallization. The optimum activation temperature for CB is 750 °C. The recrystallization reaction at 900 °C is not conducive to improving the reactivity.
2. Under room and low-temperature modification conditions, low purity CB is not an ideal SCM, and the presence of impurities leads to a decrease in strength. Only the CB modified at 750 °C fulfilled the Chinese standard, and its activity was still lower than that of the control group.
3. The results of the alkali-activated tests differ, with low purity CB showing good compatibility with the AAC. At 750 °C, CB can replace parts of the slag without causing a loss of strength. The addition of CB increased the strength, and a difference in pozzolanic activity was observed at 150 °C. It is likely that low-temperature calcination enhances the water absorption and ion exchange capacities of CB, reduces the water content, increases the concentration of the alkali solution, and improves the rate and degree of hydration.
4. At low temperatures, fly ash particles were involved in the hydration reaction, and leaf-like C-S-H gels were found in its hydration pores, which played a role in hydration filling. At high temperatures, intact fly ash particles, which did not participate in the hydration reaction and only acted as fillers, were observed, and flower-like C(N)-S-H gels coexisted inside. Due to the fact that CB absorbs water, the amplitude of the crystalline band decreases, whereas the degree of hydration increases. Meanwhile, dehydroxylation increased the Si/Al ratio, which shifted the position of the peaks.

## Figures and Tables

**Table 1:** Chemical composition of the main mineral materials.

Raw Materials	CaO	SiO_2_	Al_2_O_3_	SO_3_	MgO	Fe_2_O_3_	TiO_2_	K_2_O	LOI
Cement	62.50	20.44	4.67	2.86	2.53	3.25	-	0.53	1.98
Slag	39.3	34.53	12.73	0.4	8.27	1.93	0.9	0.8	0.3
Fly ash	4.8	56.2	22.5	1.02	1.56	5.09	1.8	0.9	3.8
CB	1.62	71.2	13	-	2.71	0.75	0.1	1.01	7.35

## Data Availability

The metadata of this work are available from the corresponding author upon reasonable request.
